# Familial Risk Factors in Thyroid Cancer Across Generations and Geographics: A Systematic Review and Meta-Analysis

**DOI:** 10.3390/curroncol32120712

**Published:** 2025-12-17

**Authors:** Madeleine B. Landau, Natalie J. Mikhailov, Amreena Singh, Ebtihag O. Alenzi, Baraah Abu Alsel, Mohammed M. Ismail, Manal S. Fawzy, Eman A. Toraih

**Affiliations:** 1 School of Medicine, Tulane University, New Orleans, LA 70112, USA; mlandau@tulane.edu (M.B.L.); nmikhailov@tulane.edu (N.J.M.); asingh9@tulane.edu (A.S.); 2 Family and Community Medicine Department, College of Medicine, Princess Nourah bint Abdulrahman University, P.O. Box 84428, Riyadh 11671, Saudi Arabia; eoalenzi@pnu.edu.sa; 3 Medical Sciences & Preparatory Year Department, North Private College of Nursing, Arar 73244, Saudi Arabia; baraahsel@nec.edu.sa; 4 Department of Anatomy, Faculty of Medicine, Northern Border University, Arar 91431, Saudi Arabia; 2351357609@nbu.edu.sa; 5 Center for Health Research, Northern Border University, Arar 73213, Saudi Arabia; 6 Department of Cardiovascular Perfusion, Interprofessional Research, College of Health Professions, SUNY Upstate Medical University, New York, NY 13210, USA; 7 Genetics Unit, Department of Histology and Cell Biology, Faculty of Medicine, Suez Canal University, Ismailia 41522, Egypt

**Keywords:** thyroid cancer, family history, genetics, geographic trends, relative risk, systematic review, meta-analysis

## Abstract

The increasing global incidence of thyroid cancer highlights the importance of accurately assessing risk factors, particularly those related to family history. Although having affected family members is widely recognized as a risk factor for thyroid cancer, the exact degree of risk and its variation across types of familial relationships, parental gender, and geographic regions remain unclear. This systematic review and meta-analysis aimed to clarify the association between family history and thyroid cancer risk. We conducted a comprehensive literature search of PubMed, Web of Science, and Embase following PRISMA guidelines, identifying 13 studies from 503 initially screened. Statistical analyses were performed using random-effects models to estimate pooled odds ratios and risk ratios, with subgroup analyses to assess variations across population and relationship types. Our findings showed an approximately 4.5-fold higher risk of thyroid cancer in individuals with affected family members. Individuals with affected siblings were more likely to develop thyroid cancer while the risks associated with maternal and paternal family history were comparable in magnitude, with no statistical difference between them. Socioeconomic, educational, and lifestyle differences did not significantly influence risk, and geographic variations in familial risk could not be statistically confirmed by the subgroup analysis, in the context of high between-study heterogeneity. These results suggest that family history is a substantial risk factor for thyroid cancer, reinforcing the need for enhanced surveillance and screening strategies for those with a familial predisposition.

## Simple Summary

Thyroid cancer rates are increasing worldwide, and having family members with thyroid cancer may raise a person’s risk of developing the disease. While it is known that family history is important, we did not know exactly how much risk was linked to different types of family relationships or whether it varied by country. In this study, we reviewed and combined data from published studies to see how a family history of thyroid cancer affects risk. We found that people with affected family members, especially siblings, are much more likely to develop thyroid cancer themselves. The risk was similar whether the family history came from the mother or father, and differences between countries were not significant. These results suggest that people with a family history of thyroid cancer may benefit from earlier or more frequent health checks, which could help with early detection and better outcomes.

## 1. Introduction

Thyroid cancer has emerged as a significant global health concern, representing one of the most prominent and rapidly increasing cancer diagnoses worldwide [1,2]. According to recent World Health Organization estimates, the global burden reached 821,214 new cases in 2022, with an age-standardized incidence rate (ASIR) of 9.1 per 100,000 people [3,4], reflecting a substantial public health challenge that spans diverse geographic and demographic boundaries. This rising incidence pattern, however, presents a complex epidemiological picture, with some regions showing continued increases. In contrast, others, notably the United States, have demonstrated a modest decline of approximately 2% annually since 2014—a trend largely attributed to the implementation of more stringent diagnostic criteria [5].

Advancements in medical imaging technologies, such as high-resolution ultrasonography and advanced cross-sectional imaging, have fundamentally altered the landscape of thyroid cancer detection [6,7]. However, this technological progress has introduced new challenges in distinguishing clinically significant malignancies from incidental findings, necessitating a more nuanced understanding of risk factors to guide screening and diagnostic protocols [7,8]. Within this context, the role of hereditary factors has gained increasing prominence [9], although their precise contribution to disease risk remains incompletely characterized.

Current evidence has established several well-documented risk factors for thyroid cancer, including exposure to ionizing radiation, female sex, and iodine deficiency [10,11,12,13]. Additionally, specific genetic syndromes, such as multiple endocrine neoplasia type 2 (MEN2), familial adenomatous polyposis (FAP), and Cowden syndrome, have been clearly associated with thyroid malignancy [14]. However, the broader implications of family history, particularly in cases without identified genetic syndromes, are less well understood across different populations and familial relationships.

Recent investigations have suggested that family history may exert a more substantial influence on both disease risk and progression than previously recognized [15]. While several studies have documented associations between family history and more aggressive disease phenotypes, the relationship between familial patterns and thyroid cancer development has not been systematically evaluated across diverse populations and varying degrees of familial relationships [16,17,18]. This knowledge gap is particularly noteworthy given the potential implications for risk stratification and clinical management strategies.

The present study addresses these critical questions through a comprehensive meta-analysis of the available evidence. Our investigation specifically examines (1) the quantitative relationship between family history and thyroid cancer risk, (2) potential variations in risk based on the degree of familial relationship, (3) the differential impact of maternal versus paternal history, and (4) geographic variations in familial risk patterns. By synthesizing data from diverse study populations, this analysis provides crucial insights into the heritable aspects of thyroid cancer risk and implications for developing risk assessment protocols and optimizing surveillance strategies for high-risk populations. Furthermore, our findings contribute to a broader understanding of thyroid cancer etiology, potentially informing future research on both genetic and environmental risk factors.

## 2. Materials and Methods

### 2.1. Study Design and Search Strategy

This study was developed and accomplished in accordance with the most recent Preferred Reporting Items for Systematic Reviews and Meta-Analyses (PRISMA) guidelines [19]. The registration number of the related protocol is “INPLASY202560025” (https://inplasy.com/). A systematic literature search across PubMed, Web of Science, and Embase was conducted on 17 August 2024, to identify the extent of peer-reviewed literature on demographic and lifestyle factors related to the heritable risk of developing thyroid cancer. To collect as many relevant studies as possible, a broad search was conducted utilizing only the following search terms: ((Lifestyle) OR (Genetics) OR (Epigenetics) OR (Benign Thyroid Conditions)) AND (Family History) AND (Thyroid Cancer).

### 2.2. Study Selection

The articles retrieved from these databases were screened against the inclusion criteria. Articles included in the study were those that described (i) a study cohort containing patients with a family history of thyroid cancer, (ii) both a group of patients without thyroid cancer and a group of patients with thyroid cancer, and (iii) reported relevant outcomes of interest. Outcomes of interest included demographic characteristics (age, sex, income, education, marital status), lifestyle factors (body mass index (BMI), alcohol consumption, smoking status, previous radiation exposure, benign thyroid conditions), and information on family members with thyroid cancer (father, mother, parent, sibling, child, 1st degree relative, other relative). Studies were excluded if they were not peer-reviewed and were designed as case reports/series, letters to the editor, editorial comments, reviews, meta-analyses, or written in a non-English language. Additionally, studies that lacked either a non-thyroid cancer group or evidence of family history of thyroid cancer were marked as ineligible. All articles underwent an initial screening based solely on title and abstract, followed by full-text review.

### 2.3. Data Extraction

All articles that were found to be eligible based on the inclusion and exclusion criteria proceeded to data extraction. Two independent investigators each independently extracted the outcomes of interest (N.M., M.B.L.). The extracted data was verified by a third investigator (A.S.). No automation tools were used at any point. Primary outcomes of interest were demographic characteristics, such as age and sex distribution of the study population, and the relationship between patients with/without thyroid cancer and the relative who was diagnosed with thyroid cancer. Other demographic characteristics (income, education, marital status) and lifestyle factors (BMI, alcohol consumption, smoking status, previous radiation exposure) were also included. Benign thyroid conditions, including goiter, benign thyroid nodules, hypothyroidism, and hyperthyroidism, were also accounted for as secondary outcomes of interest.

Age-stratified analyses were conducted to evaluate differences in familial risk among early-onset and later-onset thyroid cancer cases. We used <45 years old as the cutoff for early-onset, as this threshold has been widely applied in previous epidemiological studies and was used in the American Joint Committee on Cancer (AJCC) staging system [20,21].

Income level was categorized into low, middle, and high using the original categories from each study, reflecting each region’s census or index-based classification rather than a standardized global theme.

### 2.4. Statistical Analysis

All data analyses were performed using RStudio Build (version 2024.09). A single-arm meta-analysis was performed for demographic characteristics to generate pooled estimates of mean raw data (MRAW) or untransformed proportions with 95% confidence intervals (CI) were reported for the estimated pooled results from studies. For pairwise comparisons, estimates of mean difference served as quantitative measures of the strength of evidence, which were then converted to odds ratios (ORs) or relative risks (RRs) with 95% CIs for better interpretation within clinical domains. Given the inherent diversity in study populations, geographic locations, and study designs spanning several decades, a random-effects model was selected. Sub-analyses were conducted that incorporated geographic variation, degree of relatedness, and the differential impact of maternal vs. paternal family history. Publication bias was assessed using a funnel plot and Egger’s test. The Mantel-Haenszel and DerSimonian-Laird estimators were employed to calculate chi-square values and to adjust for heterogeneity. The *p*-value threshold for rejecting the null hypothesis was set at *p* < 0.05.

This manuscript was edited for clarity and readability using the Claude GenAI tool (Anthropic) and Grammarly. All scientific ideas, methodology, data collection, analysis, and interpretation were performed entirely by the authors.

## 3. Results

### 3.1. Eligible Studies

**Figure 1:**
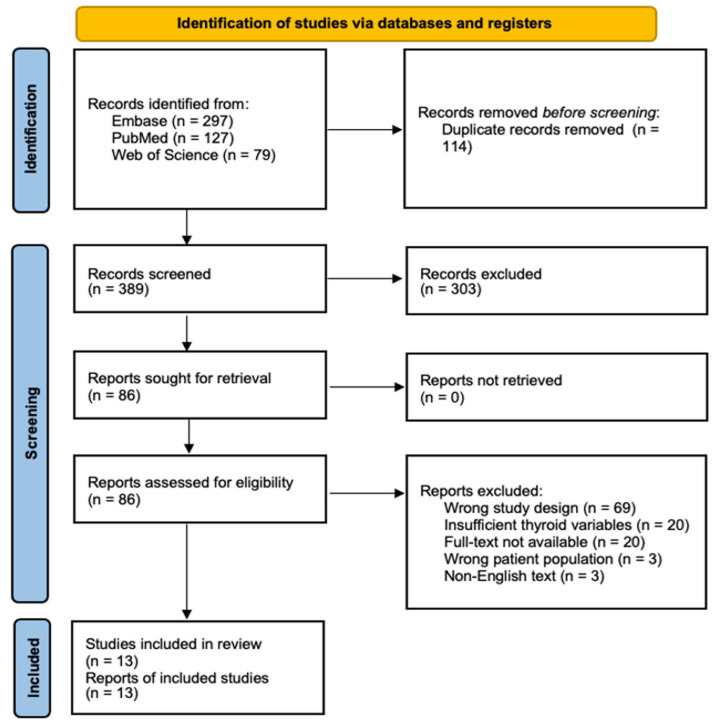
PRISMA Flow Diagram of Study Selection Process. Systematic search and selection process following PRISMA guidelines. An initial database search yielded 503 records, of which 13 studies met the final inclusion criteria after duplicate removal, screening, and eligibility assessment.

Our systematic literature search initially identified 503 potentially relevant studies across 3 major databases (PubMed, Web of Science, and Embase). Following duplicate removal (n = 114) and screening against inclusion criteria, 13 studies met the eligibility requirements for quantitative synthesis (Figure 1).

The included studies comprised 11 case-control and 2 cross-sectional designs, spanning three decades (1987–2021) and four geographic regions (Asia, America, Europe, and Oceania). The cumulative sample size included 5398 thyroid cancer cases and 204,639 controls, with individual study populations ranging from 293 to 200,576 participants (Table 1).

### 3.2. Demographic Characteristics of the Study Population

#### 3.2.1. Age Distribution

**Figure 2:**
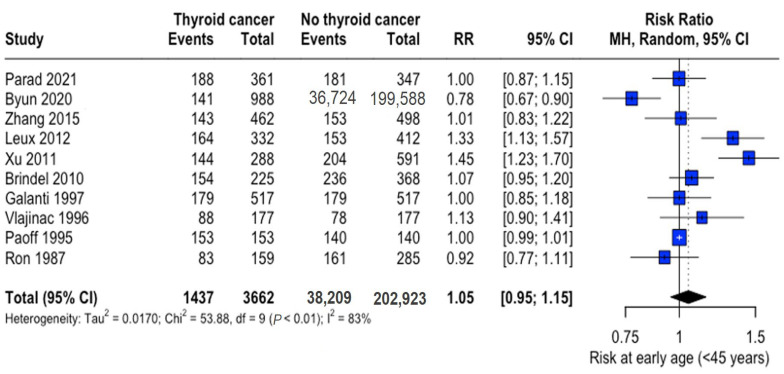
Age-related risk of thyroid cancer. Forest plot showing relative risk (RR) of thyroid cancer in early-onset (<45 years) versus later-onset cases. Central diamonds represent pooled estimates; horizontal lines indicate 95% confidence intervals [22,23,25,26,27,28,31,32,33,34].

The mean age range was 29.3–53.2 years (thyroid cancer group) and 29.6–54.2 years (control group), with thyroid cancer patients being marginally younger overall (mean difference: -3.10 years, 95% CI: −6.15 to −0.05). Analysis of early-onset cases (<45 years) revealed no significant difference in risk (RR: 1.05, 95% CI: 0.95–1.15) (Figure 2).

#### 3.2.2. Sex Distribution

**Figure 3:**
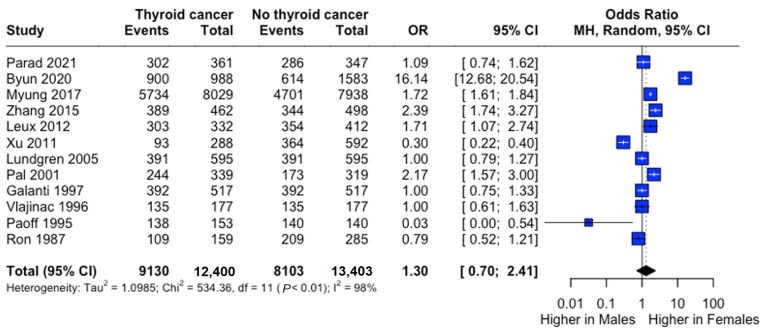
Sex-specific risk of thyroid cancer. Forest plot comparing thyroid cancer risk between males and females. Odds ratio (OR) and 95% confidence intervals are shown. Central diamonds represent pooled estimates; horizontal lines indicate 95% confidence intervals [22,23,24,25,26,27,29,30,31,32,33,34].

Female predominance was observed in both groups (thyroid cancer: 73.9%; controls: 61.9%). Males demonstrated significantly lower risk (RR: 0.73, 95% CI: 0.55–0.98), though odds ratio analysis suggested a non-significant trend toward higher female risk (OR: 1.30, 95% CI: 0.70–2.41) (Figure 3).

#### 3.2.3. Socioeconomic Factors

**Figure 4:**
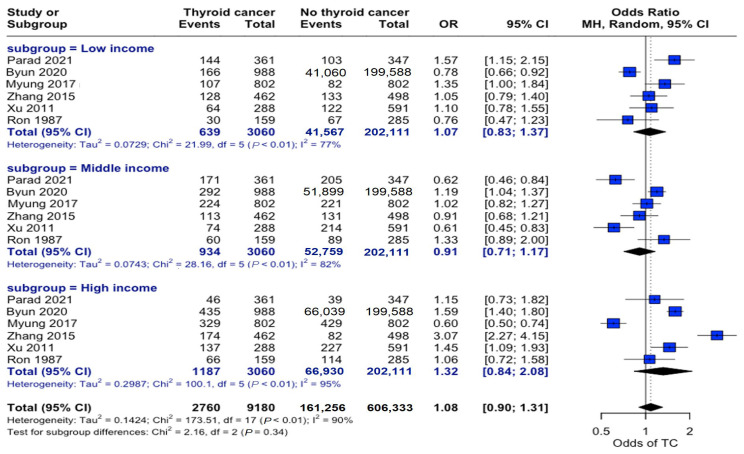
Income-stratified risk analysis. Forest plot showing odds ratios for thyroid cancer risk across three income categories. Horizontal lines represent odds ratios (ORs) and 95% confidence intervals (CIs). Categorizing individuals by income level did not demonstrate a significant relationship between socioeconomic status and thyroid cancer risk [22,23,24,25,27,34].

Analysis across income strata revealed no significant associations. Low-income (OR: 1.07, 95% CI: 0.83–1.37), middle-income (OR: 0.91, 95% CI: 0.71–1.17), and high-income (OR: 1.32, 95% CI: 0.84–2.08) individuals showed no differential association in propensity to develop this disease (Figure 4).

**Figure 5:**
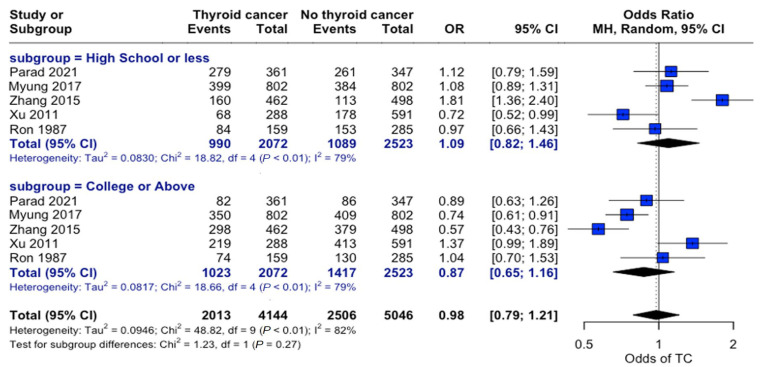
Education-related risk analysis. Forest plot comparing thyroid cancer risk stratified by educational attainment. Horizontal lines represent the odds ratio (OR) and 95% confidence intervals. There was no significant association between education level and thyroid cancer risk (OR: 0.98, 95% CI: 0.79–1.21) [22,24,25,27,34].

Examining education levels in thyroid cancer showed no significant overall association between education level and thyroid cancer risk (OR = 0.98, 95% CI: 0.79–1.21). When individuals were grouped by “High School or Less” and “College or Above” education levels, there were no significant differences in the odds of developing thyroid cancer across the strata (Figure 5).

#### 3.2.4. Marital Status

**Figure 6:**
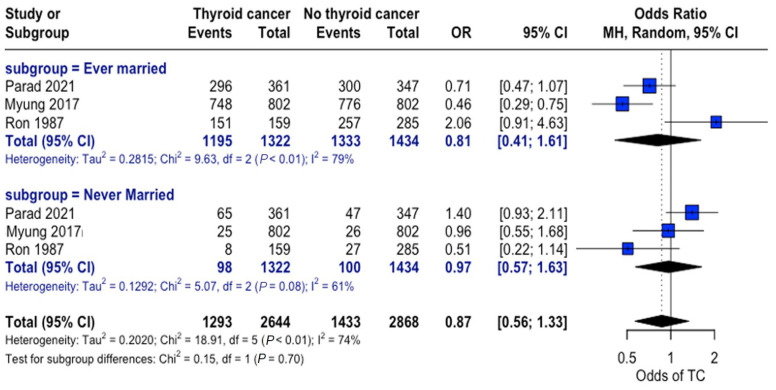
Marital status and thyroid cancer risk. Forest plot showing odds of developing thyroid cancer based on an individual’s marital status for the thyroid cancer and control groups [22,24,34].

When grouped by marital status (ever married vs. never married), a higher proportion of individuals were found to be married in both the thyroid cancer and control groups. Nonetheless, no consistent significant differences were observed between the thyroid cancer and no thyroid cancer groups across studies, and no significant overall association was observed between marital status and thyroid cancer risk (Figure 6).

### 3.3. Lifestyle and Thyroid-Related Comorbidities

**Figure 7:**
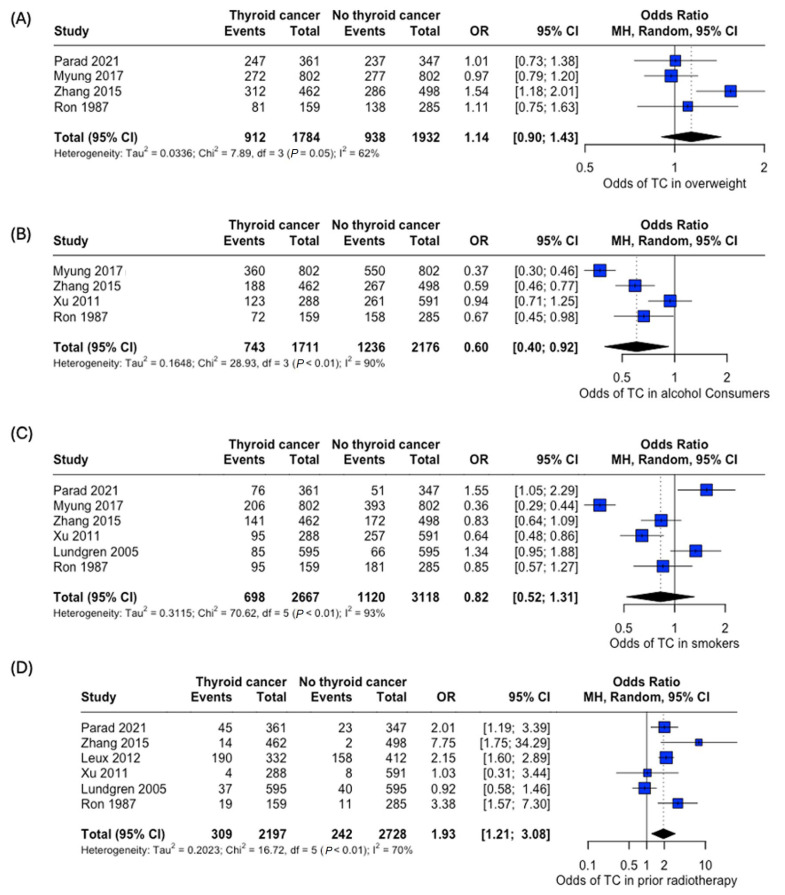
Odds of developing thyroid cancer based on lifestyle factors and comorbidities. Odds of thyroid cancer in (**A**) Overweight, (**B**) Alcohol consumers, (**C**) Smokers, and (**D**) Patients with prior radiotherapy [22,24,25,26,27,29,34].

**Figure 8:**
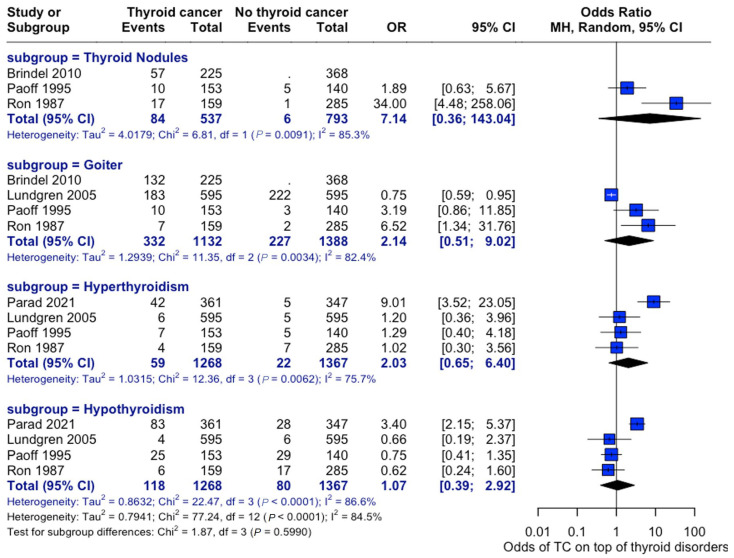
Thyroid-Related Disorders and Cancer Risk. Forest plot showing associations between pre-existing thyroid conditions and subsequent cancer risk—analysis stratified by condition type [22,28,29,33,34].

An analysis of the risk of developing thyroid cancer demonstrated no significant difference in terms of association with comorbidities. When collected, data on overweight status (OR: 1.14, 95% CI: 0.90–1.43), alcohol consumption (OR: 0.60, 95% CI:0.40–0.92), and smoking habits (OR: 0.82, 95% CI: 0.52–1.31) were all insignificant, while the case for prior radiotherapy was identified to be statistically significant (OR: 1.93, 95% CI: 1.21–3.08) (Figure 7). Patient history of other thyroid-related conditions or disorders showed no link with the development of thyroid cancer (Figure 8).

### 3.4. Association Between Family History of Thyroid Cancer and Thyroid Cancer Risk

#### 3.4.1. Overall Analysis

**Figure 9:**
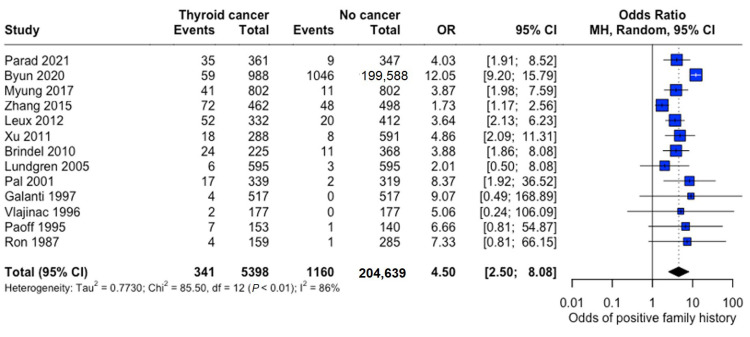
Overall Family History Association. Forest plot showing pooled and study-specific odds ratios for thyroid cancer risk associated with positive family history. A random-effects model was used due to significant heterogeneity (I^2^ > 50%) [22,23,24,25,26,27,28,29,30,31,32,33,34].

Family history demonstrated a strong association with thyroid cancer risk (pooled OR: 4.50, 95% CI: 2.50–8.08), indicating substantially 4.5 times higher odds among individuals with affected family members (Figure 9).

#### 3.4.2. Geographic Variations

**Figure 10:**
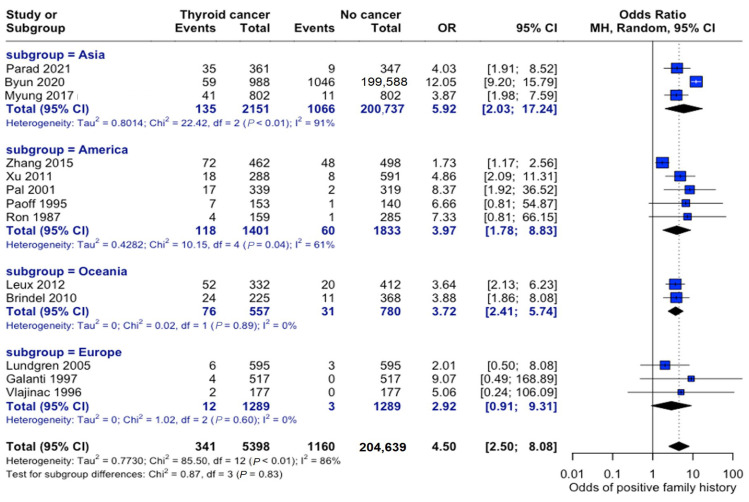
Geographic variation in family history risk. Forest plot showing odds ratios stratified by geographic region. Markers’ sizes are proportional to study weight; heterogeneity statistics are provided for each [22,23,24,25,26,27,28,29,30,31,32,33,34].

The association between family history and thyroid cancer risk varied across different geographic regions. Studies originating in Asia consisted of study populations that were nearly six times more likely to develop thyroid cancer if a family history of thyroid cancer was present (OR: 5.92, 95% CI: 2.03–17.24). Studies conducted in Europe showed the weakest association between these variables (OR: 2.92, 95% CI: 0.91–9.31), although patients were still roughly three times more likely to develop thyroid cancer if they had a family member diagnosed with thyroid cancer. The test for subgroup differences by continent was not statistically significant; however, overall heterogeneity in the meta--analysis was high (I^2^ = 83%), indicating that true effect sizes likely vary across studies. Thus, geographic variation could not be statistically confirmed by this subgroup analysis, which may have been underpowered to detect modest regional differences or other unmeasured sources of variability (Figure 10).

#### 3.4.3. Degree of Relatedness

**Figure 11:**
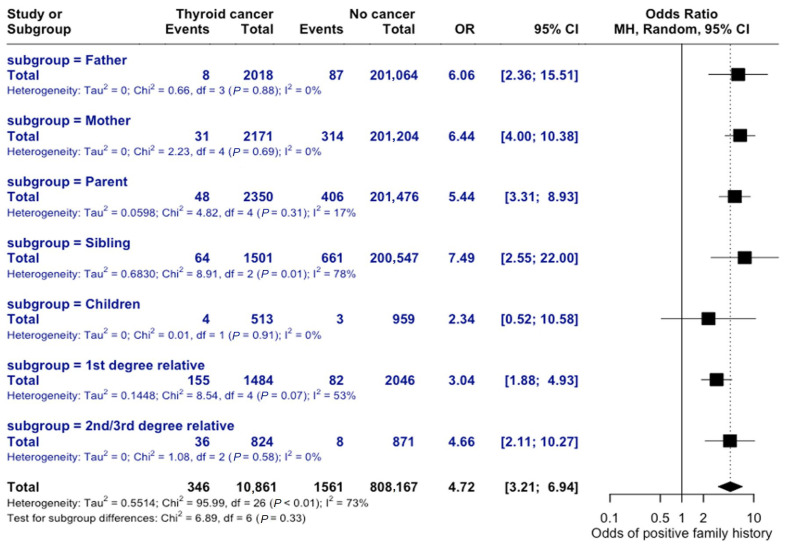
Risk by Degree of Relatedness. Forest plot comparing odds ratios across different familial relationships. Subgroup analysis shows non-significant differences (*p* = 0.33).

The risk of thyroid cancer varies based on the degree of relatedness in family history. If the relative was previously diagnosed with thyroid cancer, the odds of an individual developing thyroid cancer were 6 times greater than those without a family history (OR = 6.06, 95% CI = 2.37–15.51). A family history due to a child’s diagnosis resulted in the lowest familial odds of developing thyroid cancer, OR = 2.34 (95% CI = 0.52–10.58), while family history through siblings with prior thyroid cancer diagnosis had the greatest association (OR = 7.49, 95% CI = 2.55–22.0), as seen in Figure 11. The test for subgroup differences was not statistically significant, suggesting that the observed differences in risk by degree of relatedness may not be statistically meaningful.

#### 3.4.4. Differential Impact of Paternal vs. Maternal Family History

**Figure 12:**
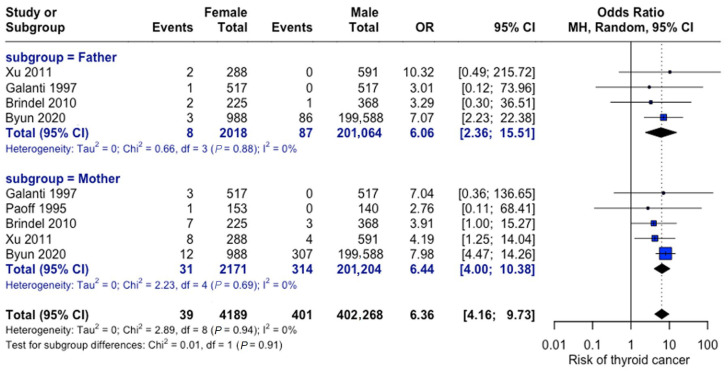
Parental history comparison. Forest plot comparing maternal versus paternal family history effects. Analysis shows consistent findings across studies for both parental categories [23,27,28,31,33].

As depicted in Figure 12, maternal versus paternal history showed comparable risks. The odds of thyroid cancer were six-fold in patients with a positive family history attributable to the mother (OR = 6.44, 95% CI: 4.0–10.38) and the father (OR = 6.06, 95% CI: 2.37–15.51). No significant difference was observed between parental effects.

## 4. Discussion

This comprehensive meta-analysis provides novel insights into the relationship between family history and thyroid cancer risk across diverse populations and familial relationships. Most notably, individuals with a family history of thyroid cancer demonstrated approximately 4.5-fold higher risk compared to those without such a history. This magnitude of excess risk is broadly consistent with large registry-based and case–control studies that have reported substantially elevated risks of thyroid cancer among first-degree relatives, while our review extends this evidence by formally synthesizing estimates across continents and by relationship type. This association persisted across geographic regions and familial relationships, with sibling history conferring the highest risk, followed by parental history. The comparable risks observed between maternal and paternal history suggest that genetic factors may play a more significant role than parent-specific environmental, epigenetic, or lifestyle influences [35].

A previous European cohort study focused exclusively on lifestyle factors, such as diet, exercise, smoking, and alcohol use, as contributors to thyroid cancer [36]. However, the relative risks of family history presented in this work highlight that the isolated contribution of heritable genetic risk still strongly outweighs the risks of factors that can be modified through lifestyle improvements.

The elevated familial risks observed in our meta-analysis align with growing evidence that a subset of non-medullary thyroid cancers arises on a hereditary background [37]. Familial non-medullary thyroid carcinoma is estimated to account for approximately 3–9% of all thyroid cancer cases [38]. It appears to follow an autosomal dominant pattern with incomplete penetrance and substantial genetic heterogeneity. Several studies have implicated germline variants and susceptibility loci in genes involved in DNA damage response and other pathways (for example, *CHEK2*, *ATM*, *BRCA2,* and additional candidate predisposition genes identified by next-generation sequencing), although no single high-penetrance gene has been established as the predominant driver of familial non-medullary thyroid cancer [39,40,41,42]. It is important to note that the primary studies included in this meta-analysis did not report individual-level germline genetic data; therefore, the genetic aspects discussed here draw on external literature and cannot be directly evaluated within our dataset.

The observed geographic variations in risk, though not statistically significant, merit careful consideration in the context of healthcare accessibility and screening practices. Asian populations demonstrated the strongest familial associations, while European cohorts showed more modest effects. These differences may reflect variations in genetic backgrounds, environmental exposures, or healthcare system characteristics rather than true biological differences in familial risk. The similar risk levels for maternal and paternal histories suggest that screening recommendations should not differ by parental gender. However, healthcare providers should consider the cumulative impact of multiple affected family members when assessing individual risk.

These findings have important implications for clinical practice, particularly regarding risk assessment and surveillance strategies. Family history should be an essential component of thyroid cancer risk evaluation, with careful documentation of the degree of relatedness before further diagnostic workup. Enhanced screening protocols may be warranted for individuals with affected first-degree relatives, though such recommendations must account for regional variations in healthcare accessibility. The availability and cost of genetic testing may influence risk assessment strategies, particularly in rural and underserved populations, where access to specialized healthcare services may be limited.

Several methodological aspects warrant consideration when interpreting these results. The predominance of retrospective designs limits causal inference, while the temporal scope spans significant advances in diagnostic capabilities. The relatively small sample sizes in some subgroup analyses led to wide confidence intervals, particularly in the paternal history subgroup. The geographic distribution of studies may not fully represent global population diversity, and potential recall bias in self-reported family histories must be acknowledged. However, the consistency of findings across studies, as shown in the cross-sectional analysis where expert clinicians verified family history, supports the reliability of our conclusions.

These limitations suggest several priority areas for future research. Prospective cohort studies are needed to validate risk estimates and investigate age-specific risk patterns. In such studies, a detailed account of TC histopathological subtype could further inform the role of family history in TC risk. A more detailed analysis of genetic-environmental interactions could help elucidate the mechanisms underlying familial risk transmission, particularly for specific genetic variants and environmental modifiers. Additionally, research examining the effectiveness and cost-efficiency of risk-based screening protocols could inform the development of more targeted surveillance strategies.

The robust association between familial history and thyroid cancer risk emphasizes the necessity of incorporating thyroid cancer-specific family history into routine clinical evaluations. Comprehensive risk assessment protocols can facilitate early diagnosis and intervention, ultimately improving patient outcomes. Future work should focus on understanding geographic trends by examining patient cohorts with greater regional variation, particularly regarding factors influencing healthcare accessibility and the availability of genetic testing.

Taken together, these findings reinforce the need for clinical guidelines and public health policies that explicitly incorporate detailed family history into risk-stratification algorithms while avoiding over-screening in lower-risk groups. Future research should integrate detailed familial data with germline genetic testing, environmental exposure assessment, and health--system variables to clarify mechanisms of familial aggregation and to evaluate the cost--effectiveness and equity impacts of risk--adapted screening strategies across regions.

## 5. Conclusions

The findings of this meta-analysis support the need for enhanced surveillance among individuals with a family history of thyroid cancer, regardless of whether the affected relative is maternal or paternal. The consistent association observed across diverse populations suggests that genetic factors are central to disease risk. However, further research is warranted to clarify the influence of environmental and healthcare system factors. Introducing routine screening protocols for families at risk may help achieve earlier diagnosis and improve patient outcomes, especially if coupled with broader access to genetic counseling and specialized healthcare services.

## Figures and Tables

**Table 1:** Characteristics of Included Studies.

Author [Ref]	Year	Geographic Region	Study Design	Sample Size	
				Thyroid Cancer	Non-Cancer
Parad et al. [22]	2021	Asia	Case-Control	361	347
Byun et al. [23]	2020	Asia	Cross-Sectional	988	199,588
Myung et al. [24]	2017	Asia	Case-Control	802	802
Zhang et al. [25]	2015	America	Case-Control	462	498
Leux et al. [26]	2012	Oceania	Case-Control	332	412
Xu et al. [27]	2011	America	Case-Control	288	591
Brindel et al. [28]	2010	Oceania	Case-Control	225	368
Lundgren [29]	2005	Europe	Case-Control	595	595
Pal et al. [30]	2001	America	Case-Control	339	319
Galanti et al. [31]	1997	Europe	Case-Control	517	517
Vlajinac et al. [32]	1996	Europe	Case-Control	177	177
Paoff et al. [33]	1995	America	Case-Control	153	140
Ron et al. [34]	1987	America	Case-Control	159	285

## Data Availability

All data were sourced from previously published articles.

## Abbreviations

The following abbreviations are used in this manuscript:

ASIR	Age-standardized incidence rate
BMI	Body mass index
CI	Confidence interval
FAP	Familial adenomatous polyposis
MEN2	Multiple endocrine neoplasia type 2
OR	Odds ratio
PRISMA	Preferred Reporting Items for Systematic Reviews and Meta-Analyses
RR	Relative risk
TC	Thyroid cancer
WHO	World Health Organization
